# Targeted Preoperative Nutritional Support within an Enhanced Recovery After Surgery Program for Malnourished Colorectal Cancer Patients: Postoperative Outcomes Comparable to Well-Nourished Patients in a Prospective Single-Center Cohort

**DOI:** 10.1245/s10434-025-18778-5

**Published:** 2026-03-13

**Authors:** Nuria Valdés, Lucia Díaz, Patricia Torrico, Ivana Crespo, Lucrecia Mendoza, Ana Belén Ferreiro, Amparo Carrocera, María Riestra, Pilar Monge, Brenda Veiguela, Gala Gutiérrez, Marta Diéguez, Javier Albaladejo

**Affiliations:** 1 Endocrinology and Nutrition Department, Hospital Universitario Cruces, Barakaldo, Bizkaia Spain; 2 Biobizkaia Health Research Institute, University of the Basque Country, CIBERDEM, CIBERER, Endo-ERN, Barakaldo, Bizkaia Spain; 3 Endocrinology and Nutrition Department, Hospital Universitario Cabueñes, Gijón, Spain; 4 Anesthesiology and Reanimation Department, Hospital Universitario Cabueñes, Gijón, Spain; 5 Rehabilitation Department, Hospital Universitario Cabueñes, Gijón, Spain; 6 General Surgery Department, Hospital Universitario Cabueñes, Gijón, Spain

**Keywords:** Nutritional screening, Enhanced Recovery After Surgery (ERAS), Colorectal cancer, Postoperative complications, Length of stay, Malnutrition, Nutritional therapy, Perioperative nutrition

## Abstract

**Background:**

Malnutrition is associated with poor surgical outcomes, but evidence supporting targeted nutritional intervention within multimodal pathways remains limited. We assessed whether preoperative nutritional support, delivered selectively to malnourished colorectal cancer patients within an Enhanced Recovery After Surgery (ERAS) framework, was associated with postoperative outcomes comparable to those of well-nourished patients.

**Methods:**

We conducted a prospective observational cohort study in an ERAS-certified Spanish hospital between January 2020 and December 2021. A total of 187 consecutive adult patients undergoing elective primary colorectal cancer resection were screened for malnutrition by using Malnutrition Universal Screening Tool and Global Leadership Initiative on Malnutrition criteria. Malnourished patients received individualized nutritional counseling and oral nutritional supplements ≥ 7 days preoperatively, per ESPEN guidelines. Primary outcomes were length of stay (LOS) and 30-day postoperative complications. Multivariable logistic and Cox regression models adjusted for age, sex, body mass index, American Society of Anesthesiologists class, and nutritional status.

**Results:**

The cohort’s mean age was 69.3 ± 9.6 years, 62.6% were male, and 42 (22.4%) were diagnosed with malnutrition. No significant differences were observed in complication rates (38.1% vs. 31.7%, *P* = 0.276) or LOS (median 8 vs. 7 days, *P* = 0.36) between malnourished and well-nourished groups. In multivariable analysis, malnutrition was not independently associated with postoperative complications (adjusted odds ratio 1.47; 95% confidence interval 0.7–3.07; *P* = 0.31) or prolonged LOS (adjusted odds ratio 0.77; 95% confidence interval 0.53–1.12; *P* = 0.17). No 30-day deaths occurred.

**Conclusions:**

Within a structured ERAS program, targeted preoperative nutritional support was associated with postoperative outcomes that were not worse than those of well-nourished peers. These findings support a selective “screen-and-treat” nutritional strategy in routine perioperative care.

Malnutrition affects a disproportionately high proportion of oncology patients, with prevalence estimates ranging from 20% to more than 70% in large multicenter studies^1,2^. The risk is particularly pronounced in tumors of the gastrointestinal tract, head and neck, liver, and lung, where catabolic inflammation, dysphagia, or malabsorption compound reduced dietary intake.^1,3^

Colorectal cancer (CRC) is the third most diagnosed malignancy worldwide. According to GLOBOCAN 2022, an estimated 1.9 million new CRC cases and ~904,000 deaths occurred globally in 2022, accounting for about one in ten cancer diagnoses and deaths.^4^ Patients with CRC are especially vulnerable to malnutrition because tumor-induced metabolic derangements, intestinal obstruction, treatment-related toxicities, and cancer-associated anorexia converge to impair nutrient assimilation.^5^ Prevalence rises with advancing age and tumor stage; up to half of older adults with locally advanced or metastatic CRC meet diagnostic criteria for disease-related malnutrition.^6^

Malnutrition is not a benign epiphenomenon: it independently predicts prolonged hospitalization, higher rates of surgical-site infection, impaired anastomotic healing, cardiopulmonary complications, and increased postoperative mortality.^7,8^ In the perioperative setting, it also delays mobilization and functional recovery, undermining the objectives of modern surgical care.

Enhanced Recovery After Surgery (ERAS) programs are multimodal perioperative care pathways designed to reduce surgical stress, maintain physiological function, accelerate recovery, and reduce complications and mortality.^9–11^ In this sense, ERAS protocols cover the entire surgical process from diagnosis to full incorporation into daily activities after the surgery. These protocols are based on three fundamental pillars: (1) the application of a set of perioperative measures and strategies; (2) interdisciplinary care, understood as the joint and structured participation of the various healthcare professionals involved; (3) and active patient participation throughout the process. Follow ERAS protocols are effective and safe, reduce hospital stays, and are associated with fewer complications without increasing readmission rates.^10–15^

Preoperative nutritional care, which begins with nutritional screening, is one of the principles of the ERAS protocol.^10,11,16–18^ However, the strength of evidence supporting its effectiveness varies from low to moderate.

The present prospective cohort study evaluated whether, in an established ERAS framework, selective preoperative nutritional support for malnourished colorectal cancer patients is associated with postoperative outcomes comparable to those of well-nourished patients. Primary endpoints were length of hospital stay (LOS) and overall postoperative complications; secondary endpoints included 30-day readmission and mortality.

## Materials and Methods

### Study Design and Setting

A prospective, observational cohort study was conducted at an ERAS-certified secondary hospital from January 1, 2020 to December 31, 2021. The center is accredited by the Spanish Group of Multimodal Rehabilitation (GERM)^14^ and participates in the national IMPRICA implementation program.^17,18^

### Participants

Eligible patients were adults (≥ 18 years) scheduled for elective primary CRC resection within the institutional ERAS pathway. Exclusion criteria were emergency surgery (within 72 h of admission), stage IV cancer or any other synchronous primary tumors, an American Society of Anesthesiologists (ASA) physical status ≥ IV, chronic kidney disease stage 5 (creatinine > 2.83 mg/dL or on dialysis), or refusal to provide informed consent.

### Ethical Considerations

All participants provided informed consent. The study adhered to institutional guidelines and was conducted under ethical approval in accordance with the Declaration of Helsinki.

### ERAS Program

The multidisciplinary ERAS team (surgeons, anesthesiologists, endocrinologists, dietitians, physiotherapists, and specialized nurses) applies a 32-item protocol aligned with Spanish National Health Service recommendations. Compliance is audited yearly and exceeds 80% of mandatory elements.

### Nutritional Assessment and Intervention

Preoperative nutritional screening was conducted by using the Malnutrition Universal Screening Tool (MUST),^19^ which scores patients based on body mass index (BMI), unintentional weight loss, and recent reduced intake. Each risk predictor is scored from 0 to 2 points. Patients with a MUST score ≥ 1 and/or serum albumin ≤ 3.1 g/dL were further assessed by using the Global Leadership Initiative on Malnutrition (GLIM) consensus criteria to confirm malnutrition diagnosis.^20^ Patients diagnosed as malnourished received personalized nutritional counselling and oral nutritional supplements (ONS), targeting an intake of 25–30 kcal/kg/day and 1.2–1.5 g protein/kg/day, in line with ESPEN guidelines.^8,21^ Nutritional support began at least 7 days before surgery and continued for a minimum of 2 weeks postoperatively. Well-nourished patients followed standard ERAS feeding (carbohydrate loading + early oral intake).

### Data Collection

Clinical and biochemical data were collected prospectively from the medical records and through patient interview by the ERAS nurse in a specifically designed secure electronic data collection system at the national level, Castor EDC (https://www.castoredc.com) within the GERM network.^22^ This platform complies with national data protection laws. Recorded demographic and baseline variables included age, sex, BMI, smoking status, comorbidities, ASA score; laboratory parameters (albumin, glucose, hemoglobin, leukocytes and neutrophils counts, cholesterol, C-reactive protein, and hemoglobin A1c; operational details: surgical approach (open vs. laparoscopic), operative time, blood loss; and nutritional details: MUST score, GLIM category, ONS type/dose, caloric and protein intake.

### Outcomes

The primary outcomes analyzed were (1) LOS, defined as days from index operation to hospital discharge; and (2) composite postoperative morbidity—any complication graded ≥ II by Clavien–Dindo during index admission or within 30 days of surgery. The secondary outcomes assessed were specific complications that occurred during index hospitalization and within 30 days of surgery; and readmission and mortality rates at 30 days. Thirty-day mortality is defined as death within 30 days of the index operation. The day of the operation was considered as day 0 for all variables. Postoperative follow-up was conducted in person or by phone.

### Sample Size and Power

With an expected malnutrition prevalence of 20% and aiming to detect a 2-day reduction in LOS (SD ± 3), α 0.05/β 0.20, a minimum of 180 patients (≈36 malnourished) was required. The final cohort (n = 187) satisfied this threshold.

### Statistical Analysis

Continuous variables were summarized as mean ± standard deviation when normally distributed or as median (interquartile range [IQR]) when skewed; ranges are reported where informative. Categorical variables are presented as number (percentage)**.** Normality was assessed with the Kolmogorov–Smirnov test; nonnormal data were log-transformed before analysis. Group differences were examined with Student’s *t* test or Mann–Whitney *U* test for quantitative variables and with χ^2^ test or Fisher’s exact test for qualitative variables.

A binary logistic regression model was built to identify independent predictors of “any postoperative complication” within 30 days, it was adjusted for age, sex, BMI, ASA class, and nutritional status; results are expressed as adjusted odds ratios with 95% confidence intervals (CI). Covariates (age, sex, BMI, and ASA class) were prespecified based on clinical relevance; we also conducted sensitivity models adding diabetes, hypertension, and anemia. These models were not intended to establish causality but to reduce confounding in estimating associations. LOS to discharge alive was analyzed by Kaplan–Meier curves and a Cox proportional-hazards model using the same covariates; proportional-hazards assumptions were verified with Schoenfeld residuals, and results are reported as adjusted hazard ratios with 95% CI. Model calibration and discrimination were assessed with the Hosmer–Lemeshow test and c-statistic for logistic models, and with deviance residuals and Harrell’s C-index for the Cox model. All tests were two-sided, and *P* < 0.05 was considered statistically significant. Analyses were performed with IBM SPSS Statistics v29 (IBM Corp., Armonk, NY).

## Results

A total of 187 patients were included with a mean age of 69.3 ± 9.6 years (range 36–86), 90 (49.2%) were older than 70 years of age, and 117 (62.6%) were men. Mean preoperative BMI was 27.6 ± 4.94 kg/m^2^ (range 18–40); 2 patients (1.1%) were underweight (BMI < 18.5 kg/m^2^), 46 (24.6%) had normal weight (18.5–24.9), 83 (44.4%) were overweight (25–29.9), and 56 (29.9%) were obese (≥ 30). The commonest comorbidities were hypertension (47.1%), anemia (29.9%), and diabetes mellitus (17.6%); 26.7% of patients reported none. The ASA class was I in 26 patients (13.9%), II in 115 (61.5%), III in 45 (24.1%), and IV in 1 (0.5%). Clinical and biochemical data are shown in Table 1.

**Table 1:** Baseline clinical and biochemical characteristics

	Total cohort N = 187
Sex:	
Female	70 (37.4%)
Male	117 (62.6%)
Age, years	69.3 ± 9.6
Body mass index, kg/m^2^	27.6 ± 4.9
Smokers	31 (16.5%)
Hemoglobin, g/dl	12.8 ± 2.0
Leukocytes/ul	7.1 ±3.5
Neutrophils/ul	4.3 ± 1.5
Glucose, mg/dl	108.8 ± 28.8
Cholesterol, mg/dl	179.2 ± 41.6
Albumin, g/L	40.3 ± 3.7
C-reactive protein, mg/L	4.8 (IQR:1.6–10.3)
Comorbidity:	
Hypertension	87 (47.1)
Anemia	56 (29.9)
Diabetes mellitus	33 (17.6)
No	50 (26.7)
Surgical risk:	
ASA I	26 (13.9)
ASA II	115 (61.5)
ASA III	45 (24.1)
ASA IV	1 (0.5)
MUST score:	
0 (%)	140 (74.8)
1 (%)	33 (17.6)
≥ 2(%)	14 (7.4)
GLIM criteria:	
No malnutrition	145 (77.7)
Moderate malnutrition	28 (14.9)
Severe malnutrition	14 (7.4)

*ASA* American society of anesthesiologists physical status classification system; *MUST* malnutrition universal screening tool; *GLIM* global leadership initiative on malnutrition

### Nutritional Status

Recent weight loss was reported by 82 patients (43.9%) (median 4.7%, IQR 2.8–8.6%). Forty-two patients (22.4%) screened positive, 28 patients had a MUST score of 1, and 14 had a MUST score of 2 or higher. Two patients had a plasma albumin level ≤ 3.1 g/dL, one of whom had a MUST score of 1 and the other a MUST score of 2. All 42 screened-positive patients met GLIM criteria, 28 (14.9%) had moderate malnutrition, and 14 (7.4%) severe malnutrition. Baseline characteristics by nutritional group are provided in Table 2.

**Table 2:** Clinical and biochemical characteristics according to nutrition status

	Patients with malnutrition N = 42	Patients without malnutrition N = 145	*P*
Sex:			
Male	25 (59.5%)	92 (63.4%)	0.386
Female	17 (40.5%)	53 (36.6%)	
Age, years	66.3 ± 11.7	70.1 ± 8.8	0.023
Body mass index, kg/m^2^	24.9 ± 4.2	28.5 ± 4.8	< 0.001
Smokers	9 (21.4%)	22 (15.2%)	0.230
Hemoglobin, g/L	11.7 ± 2.2	13.1 ± 1.8	< 0.001
Leukocytes/ul	7.1 ± 3.7	6.9 ± 2.8	0.806
Neutrophils/ul	4.2 ± 1.2	4.7 ± 2.3	0.12
Glucose, mg/dl	107.2 ± 20.4	114.3 ± 46.8	0.16
Cholesterol, mg/dl	180.2 ± 40.9	176.0 ± 44.6	0.65
Albumin, g/L	39.1 ± 4.0	40.7 ± 3.6	0.015
C-reactive protein, mg/L	18.4 18.4 ± 29.7	7.1 ± 9.3	< 0.001
Comorbidity:			
Hypertension	14 (33.3%)	74 (51.0%)	0.032
Anemia	8 (19%)	31 (21.4%)	0.465
Diabetes mellitus	9 (21.4%)	24 (16.6%)	0.301
No	12 (28.6%)	37 (25.5%)	0.415
Surgical risk:			
ASA I	7 (16.7%)	19 (13.1%)	0.176
ASA II	22 (52.4%)	93 (64.1%)	
ASA III	12 (28.6%)	33 (22.8%)	
ASA IV	1 (2.4%)	0	

*ASA* American society of anesthesiologists physical status classification system

There were significant differences between malnourished and well-nourished patients in terms of age: 66.3 ± 11.7 years (range 36–84) vs. 70.1 ± 8.8 years (range 42–86), *P* = 0.023; BMI, 24.9 ± 4.2 kg/m^2^ (range 18–36) vs. 28.5 ± 4.8 kg/m^2^ (range 20–40), *P* < 0.001). There were no significant differences regarding sex, ASA level, or comorbidities, except for hypertension, which was less frequent among malnourished patients (33.3% vs. 51%, *P* = 0.032). Surgery was undertaken a mean time after nutrition assessment of 46.6 ± 35.9 days (range 7–181) in malnourished patients vs. 53.5 ± 45.8 days (range 5–175) in well-nourished patients, *P* = 0.376.

Regarding preoperatively analytical parameters, there were significant differences between malnourished and well-nourished patients in hemoglobin level: 11.7 ± 2.2 g/dL (range 7.1–15.7) vs. 13.1 ± 1.8 g/dL (range 7.9–16.9), *P* < 0.001; albumin level: 39.1 ± 4 g/L (range 3–47) vs. 40.7 ± 3.6 g/L (range 32–60), *P* = 0.015; and C-reactive protein: 18.4 ± 29.7 mg/L (range 0.5–128.6) vs. 7.1 ± 9.3 mg/L (range 0.5–59.1), *P* < 0.001. No significant group differences were observed for glucose, cholesterol, neutrophils, leukocytes, or hemoglobin A1c levels.

### Postoperative Outcomes

Early complications occurred in 62 of 187 patients (33.2%; 95% CI 26.8–40.2) (Table 3). Among well-nourished patients, 46 of 145 experienced early complications (31.7%; 95% CI 24.7–39.7), whereas 16 of 42 malnourished patients were affected (38.1%; 95% CI 25–53.2). The odds ratio (OR) for malnourished versus well-nourished patients was 1.32 (95% CI 0.68–2.59; *P* = 0.276).

**Table 3:** Postoperative outcomes according to malnutrition diagnoses

	Patients with malnutrition N = 42	Patients without malnutrition N = 145	*P*
Patients with any postoperative complications	16 (38.1%)	46 (31.7%)	0.275
Patients with early surgical complications	12 (28.6%)	33 (22.8%)	0.280
Patients with infection complications	5 (11.9%)	17 (11.7%)	0.580
Patients with surgical-site infection	1 (2.3%)	7 (4.8%)	0.677
Patients with medical complications	8 (19%)	16 (11%)	0.136
Patients with 1 complication	7 (16.6%)	29 (20%)	0.205
Patients with 2 complications	9 (21.4%)	15 (10.3%)	
Patients with ≥3 complications	0	2 (1.3%)	
Length of hospital stay, days	8 (IQR 5.0–15.0)	7 (IQR 5.0–11.0)	0.364
Readmission within 30 days of hospital discharge	0	3	0.592
Deaths within 30 days of hospital discharge	0	0	

The distribution of specific events likewise showed no significant differences between groups:

- Early surgical complications occurred in 12 of 42 malnourished versus 33 of 145 well-nourished patients (28.6% vs. 22.8%; OR 1.35, 95% CI 0.62–2.94; *P* = 0.28).
- Infectious complications were recorded in 5 of 42 versus 17 of 145 patients (11.9% vs. 11.7%; OR 1.01, 95% CI 0.35–2.94; *P* = 0.58).
- Surgical-site infections occurred in 1 of 42 versus 5 of 145 patients (2.4% vs. 3.4%; OR 0.68, 95% CI 0.07–6.01; *P* = 0.594).
- Medical complications were observed in 8 of 42 versus 16 of 145 patients (19% vs. 11%; OR 1.89, 95% CI 0.74–4.8; *P* = 0.316).

Univariate analysis—performed for the composite endpoint “any postoperative complication”—identified no predictor with a statistically significant association.

**Fig. 1:**
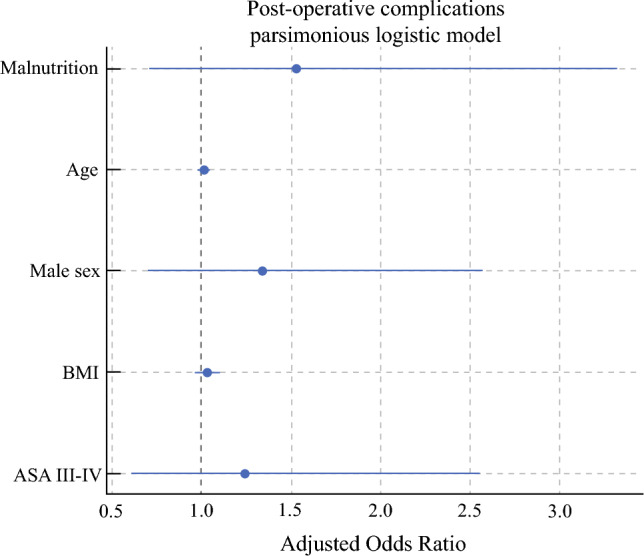
Adjusted odds ratios for “any postoperative complication” (parsimonious multivariable logistic model). Forest plot displaying the effect estimates for the five prespecified covariates—malnutrition, age, male sex, BMI and ASA class III–IV—derived from the multivariable logistic regression. Points indicate adjusted odds ratios (aOR) and horizontal bars their 95% confidence intervals; the vertical dashed line marks the null value (aOR = 1). None of the variables reached statistical significance

In the multivariable logistic model (Fig. 1), malnutrition was not independently associated with postoperative complications (adjusted odds ratio [aOR] 1.6, 95% CI 0.73–3.52; *P* = 0.24); age, male sex, BMI, and ASA class were likewise nonsignificant. The model demonstrated good calibration (Hosmer–Lemeshow χ^2^ = 6.6, *P* = 0.58) and moderate discrimination (AUC = 0.59). Adding diabetes, hypertension, and anemia to the model did not materially alter the effect estimate for malnutrition (aOR 1.72, 95% CI 0.78–3.77; *P* = 0.18), and none of the additional comorbidities reached statistical significance.

**Fig. 2:**
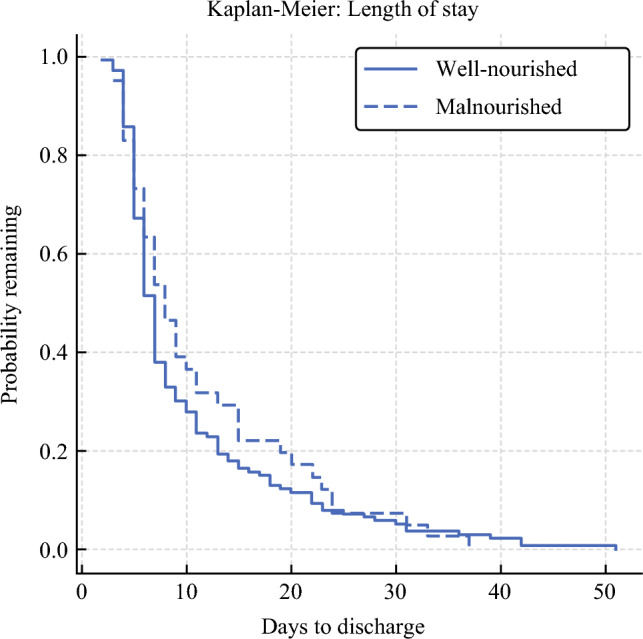
Kaplan–Meier curves for time to discharge according to nutritional status. The plot depicts the probability of remaining in hospital after colorectal-cancer surgery for well-nourished patients (solid line, *n* = 145) and malnourished patients (dashed line, *n* = 42). Median length of stay was 7 days (IQR 5–11) in the well-nourished group and 8 days (IQR 5–15) in the malnourished group. The difference between curves was not significant (log-rank *P* = 0.36)

**Fig. 3:**
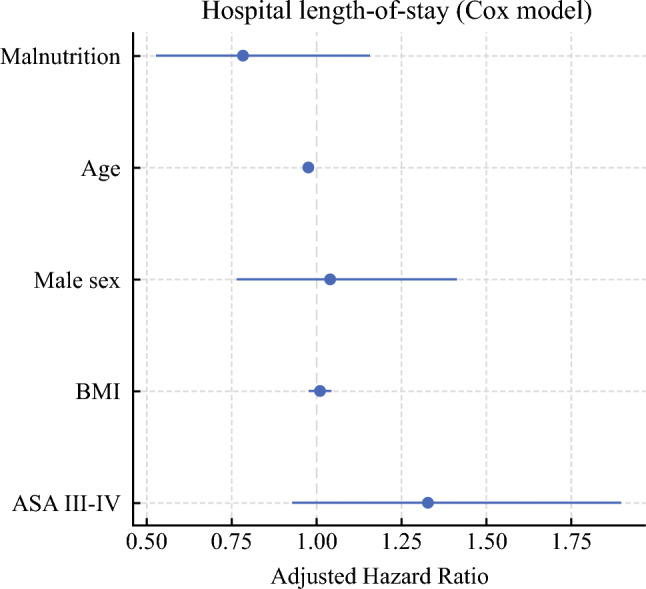
Adjusted hazard ratios for hospital length-of-stay (parsimonious Cox model). Forest plot summarising the multivariable Cox proportional-hazards analysis for time to discharge. The model includes malnutrition, age, male sex, BMI, and ASA class III–IV. Dots represent adjusted hazard ratios (aHR) and horizontal bars their 95% confidence intervals; the dashed vertical line indicates the null value (aHR = 1). Advancing age was the only independent predictor of a longer stay (aHR ≈ 0.98 per year, 95% CI 0.96–0.99)

Median LOS was 8 days (IQR 5–15) in malnourished versus 7 days (IQR 5–11) in well-nourished patients (log-rank *P* = 0.36; Fig. 2). In the parsimonious multivariable Cox model that included malnutrition, age, sex, BMI, and ASA III–IV (Fig. 3), age was an independent predictor of longer stay (adjusted hazard ratio (aHR) per additional year 0.98, 95% CI 0.96–0.99*; P* = 0.006), whereas malnutrition did not (aHR 0.78, 95% CI 0.53–1.16; *P* = 0.22). Adding diabetes, hypertension and anemia to the model did not materially alter the effect estimates for malnutrition or age, as shown in Table 4. Proportional-hazards assumptions were satisfied for all covariates (global Schoenfeld *P* = 0.42).

**Table 4:** Univariate and multivariable Cox model regression for length of hospital stay

	Univariable			Multivariable		
	HR	95% CI	*P*	HR	95% CI	P
Malnutrition	0.865	0.610–1.228	0.418	0.78	0.526–1.156	0.216
Age (per year)	0.983	0.968–0.999	0.037	0.977	0.959–0.994	0.01
Male sex	1.039	0.768–1.408	0.802	1.064	0.772–1.465	0.706
BMI (kg/m^2^)	1.01	0.980–1.040	0.520	1.013	0.978–1.049	0.464
ASA ≥3	1.187	0.841–1.675	0.329	1.336	0.933–1.912	0.113
Diabetes	0.945	0.641–1.393	0.773	0.91	0.608–1.363	0.647
Hypertension	0.952	0.709–1.277	0.743	0.947	0.663–1.354	0.766
Anemia	0.962	0.669–1.384	0.834	1.057	0.727–1.538	0.772

*BMI* body mass index; *ASA* American society of anesthesiologists physical status classification system

Readmission within 30 days occurred in three malnourished patients versus ten well-nourished patients, respectively (*P* = 0.592). No 30-day deaths were recorded in either group (Table 3).

## Discussion

This prospective ERAS-based cohort found no evidence of higher early complications or longer length of stay among malnourished patients receiving targeted support compared with well-nourished patients. Malnutrition is common among cancer patients. Previous studies have reported a wide range in the prevalence of preoperative malnutrition risk among colorectal cancer patients undergoing surgery, ranging from 12 to 47%, which aligns with the 22.4% found in our population.^23–25^ These discrepancies in reported prevalence can be attributed to the differences in tests and methods used for screening and diagnosing malnutrition. In a recent international study using GLIM phenotypic criteria (BMI and weight loss), 29.5% of patients undergoing elective surgery for colorectal cancer were classified as severely malnourished,^26^ a figure notably higher than the 7.4% found in our study. One possible explanation for this lower prevalence of severe malnutrition in our study could be the implementation of an early colorectal cancer screening program that includes patients older than 50 years and may contribute to earlier diagnosis and the inclusion of relatively healthier patients. This could also explain why malnourished patients in our population were younger than well-nourished ones—an unexpected finding given that the literature consistently reports higher malnutrition rates among patients older than 70 years,^6^ with rates ranging from 40 to 61%, and severe malnutrition affecting 10–21% of these patients.

Preoperative malnutrition is a well-established predictor of adverse surgical outcomes, including increased postoperative morbidity, mortality, and poorer oncological outcomes in gastrointestinal cancer surgery.^6,11,26^ Accordingly, ESPEN guidelines^8,21^ recommend early nutritional care, which begins with nutritional screening, to detect both overt and subtle malnutrition. This approach offers an opportunity to improve nutritional status and address specific deficiencies.

Recent interventional research is mixed. A meta-analysis by Gillis et al.^27^ emphasized the critical role of nutrition in prehabilitation, revealing that nutritional support, alone or combined with exercise, effectively reduced the length of hospital stay by 2 days following colorectal surgery. However, a Cochrane review concluded that the impact of prehabilitation (encompassing nutritional support and exercise) on reducing complications, postoperative emergency department visits, and readmissions could not be definitively established.^28^ The certainty of this evidence ranged from moderate to very low, primarily due to serious risks of bias, imprecision, and inconsistency, and the review included only three heterogeneous studies. In contrast, a newly published trimodal prehabilitation program demonstrated a significantly lower incidence of severe complications.^29^ Notably, all these studies provided prehabilitation programs, including nutritional support, to all participants, regardless of their nutritional status.

Furthermore, recent systematic reviews evaluating preoperative nutritional support for patients undergoing colorectal cancer surgery were unable to draw definitive conclusions regarding the effectiveness of oral nutritional supplements, again due to the heterogeneity of the studies reviewed.^30–32^ While oral nutritional supplements seem to provide greater benefits for patients at risk of malnutrition or those already malnourished, the limited number of studies prevents the establishment of conclusive clinical evidence.^33^ This result aligns with those of a Cochrane review of preoperative nutritional therapy in patients undergoing gastrointestinal surgery.^34^ However, most of the studies were conducted outside the framework of an ERAS program, which has itself been shown to improve hospital length of stay and reduce postoperative complications.^35,36^ Therefore, incremental gains from universal supplementation are likely to be minimal, reinforcing the need for selective intervention.

In our study, nutritional support was provided exclusively to patients diagnosed as malnourished patients, following clinical guidelines through dietary advice and oral nutritional supplements to meet their calorie and protein requirements. The goal of a preoperative nutritional program for malnourished patients is to achieve outcomes comparable to those of well-nourished patients. However, to the best of our knowledge, no previous studies have directly evaluated this approach in a comparative design.

Our findings support this hypothesis: we observed no significant differences in early postoperative complications or length of hospital stay between malnourished patients receiving nutritional support and well-nourished patients. Although an untreated malnourished group would offer the most rigorous comparison, withholding therapy is ethically untenable under current guidelines. Observational data consistently show that malnourished patients without intervention experience longer stays and more complications.^26,6^ Taken together, these data suggest that a selective screen-and-treat pathway may help to avert the excess risk typically observed, while recognizing the constraints of an observational design.

Within ERAS programs, a selective screen-and-treat pathway can be implemented with relatively modest resources—routine MUST screening, GLIM confirmation by a dietitian or nurse, brief individualized counseling, and a short preoperative and postoperative course of ONS. While a formal cost-effectiveness analysis was beyond our scope, these limited inputs could be offset by downstream savings if postoperative complications and length of stay are not increased. A pragmatic evaluation should prospectively capture program inputs (dietitian/nurse time, ONS use, clinic contacts) alongside downstream utilization (postoperative complications, readmissions, and length of stay) to estimate value. Importantly, our single-center experience in an ERAS-mature hospital may not reflect settings with different staffing, formulary access, or protocol adherence; multicenter evaluations with explicit health-economic endpoints are warranted to define generalizability and affordability.

This study has certain limitations. First, we lacked a concurrent control group of malnourished patients who did not receive nutritional therapy, so the counterfactual relies on historical evidence. Second, adherence to oral nutritional supplements was self-reported rather than objectively verified, introducing recall and social-desirability bias. Third, the work was conducted at a single center, which may limit generalizability to other hospitals and healthcare systems. In addition, as with any observational design, unmeasured and residual confounding may persist. Factors, such as detailed tumor stage beyond eligibility, frailty or sarcopenia, socioeconomic status, exact smoking exposure, inflammatory burden, surgeon-level factors, and participation in nonnutritional prehabilitation, may not have been fully captured. We prespecified and adjusted for age, sex, BMI, and ASA class and explored sensitivity models, including diabetes, hypertension, and anemia; nevertheless, residual confounding may persist. If present, such bias would plausibly disadvantage the malnourished group, suggesting that the observed similarity of outcomes is conservative.

Balancing these limitations, our study also has several strengths. First, it has a prospective design; it was conducted in a real-world clinical setting. Second, all patients were managed under a standardized ERAS protocol. Third, the number of patients enrolled exceeded that of most published studies, enabling analysis of various patient subgroups and variables with adequate statistical power. Fourth, the study’s prospective design ensured consistent data collection across all participants.

## Conclusions

Selective, guideline-driven nutritional optimization within an ERAS was associated with postoperative outcomes comparable to those of well-nourished patients in this prospective cohort. These data strengthen current ESPEN and ERAS recommendations and support routine “screen-and-treat” pathways to promote equitable surgical outcomes. Larger multicenter studies with health-economic endpoints should confirm cost-effectiveness and define the optimal duration of preoperative support.
